# Profiles of Hesitancy Toward the Herpes Zoster Vaccine Among Older Adults in China: A Latent Profile Analysis

**DOI:** 10.3390/vaccines14040331

**Published:** 2026-04-08

**Authors:** Jianing Dai, Yuanruo Xie, Yuxing Wang, Shuai Yuan, Ling Zhu, Qiang Zeng, Gang Liu, Lili You, Zhujiazi Zhang

**Affiliations:** 1 School of Health Policy and Management, Chinese Academy of Medical Sciences and Peking Union Medical College, Beijing 100080, China; daijn@student.pumc.edu.cn (J.D.); wangyx@student.pumc.edu.cn (Y.W.); 2 Department of Health Policy and Management, Mailman School of Public Health, Columbia University, New York, NY 10027, USA; yx3031@cmcu.columbia.edu; 3 Department of Health Policy and Management, Bloomberg School of Public Health, Johns Hopkins University, Baltimore, MD 21218, USA; shuai.yuan@jhu.edu; 4 Institute for Hospital Management, Tsinghua University, Shenzhen 518055, China; 5 ChongQing Nanan District Center for Disease Control and Prevention, Chongqing 400060, China; 13650502603@163.com; 6 Shenzhen Nanshan District Health Committee, Shenzhen 518052, China; email_zeng@163.com; 7 Shenzhen Center for Disease Prevention and Control, Shenzhen 518055, China; sliugang@163.com; 8 Beijing Key Laboratory of Surveillance, Early Warning and Pathogen Research on Emerging Infectious Diseases, Beijing Center for Disease Prevention and Control, Beijing 100013, China; 9 Beijing Research Center for Respiratory Infectious Diseases, Beijing 100013, China; 10 School of Public Health, Capital Medical University, Beijing 100069, China

**Keywords:** latent profile analysis (LPA), older adults in China, vaccine hesitancy, 5C model

## Abstract

**Background**: Understanding diverse psychological factors is crucial for promoting vaccination. This study focuses on psychological factors influencing HZ vaccination attitudes and intentions among older adults who have not yet received the HZ vaccine in China. **Methods**: We conducted a cross-sectional survey of 12,357 older adults (aged ≥60 years) who had not previously received the HZ vaccine. Latent Profile Analysis (LPA) was performed using the 5C psychological antecedents of vaccination (Confidence, Constraints, Calculation, Complacency, and Collective Responsibility). Multinomial logistic regression and Chi-square tests were used to identify predictors of profile membership and to investigate the nature of reported barriers. **Results**: An optimal five-profile solution was identified, with the largest group being “Willing but Obstructed” (44.6%). This profile exhibited high vaccination willingness but perceived the most significant constraints. While household income was not a direct predictor of profile membership, low-income individuals were significantly more likely to report ‘high cost’ as a primary barrier (*p* < 0.01), revealing that socioeconomic status appears to influence vaccination intention through tangible structural obstacles. **Conclusions**: Vaccination attitudes among previously unvaccinated older adults are heterogeneous. A substantial proportion are willing to be vaccinated but are hindered by socioeconomic barriers, primarily cost. Addressing the intention–behavior gap may require a shift from universal messaging to equity-focused interventions that directly address structural barriers for vulnerable groups, particularly among those who have not yet initiated vaccination.

## 1. Introduction

Herpes zoster (HZ), or shingles, represents a substantial public health burden in China, particularly for adults aged 50 and older who face a high risk of developing debilitating post-herpetic neuralgia (PHN) [1,2]. Vaccination is the most effective preventive measure. However, despite the availability of the HZ vaccine on China’s private market since 2020, coverage remains below 1% [3,4,5]. It is important to note that two main types of HZ vaccines have been introduced in China: the Changchun Bcht’s live attenuated vaccine (approved in 2023) and the GSK’s recombinant subunit vaccine (approved in 2020) [6]. These vaccines differ in their mechanisms, efficacy, and adverse event profiles. Regarding safety, while common adverse events for currently available HZ vaccines in China, particularly the GSK’s recombinant subunit vaccine, are typically mild to moderate (e.g., injection site pain, myalgia, fatigue), serious adverse events are rare [7].

While structural barriers like cost and limited provider recommendations are known contributors, they do not fully account for this low uptake [5,8,9,10,11]. Understanding vaccine hesitancy is crucial for public health efforts, especially in the context of the principle of informed consent, which emphasizes individuals’ autonomous decision-making based on comprehensive information about health interventions like vaccination [12]. A deeper understanding of the psychological antecedents to vaccination is therefore crucial. The World Health Organization’s (WHO) concept of vaccine hesitancy underscores its complex, context-specific nature [13,14]. The 5C model—encompassing Confidence, Complacency, Constraints, Calculation, and Collective responsibility—offers a robust framework for systematically examining these psychological drivers [15,16]. Although validated for other vaccines, its psychometric properties have not been established for HZ vaccination among older Chinese adults who have not yet received the HZ vaccine, a necessary prerequisite for its application [17,18,19].

Furthermore, most research has relied on variable-centered analyses (e.g., regression), which identify average associations but can mask significant population heterogeneity [5,10,11]. A person-centered approach, such as latent profile analysis (LPA), can uncover distinct psychological profiles, offering more actionable insights for targeted interventions [17,18,19]. While physician recommendation is a known driver of adult vaccination, its impact may not be uniform across different psychological profiles [10,20,21]. Understanding this interaction is critical for tailoring clinical counseling.

This study aimed to: (1) evaluate the psychometric properties of an adapted 5C scale for HZ vaccination among unvaccinated older Chinese adults; (2) identify distinct psychological profiles related to HZ vaccination attitudes and intentions among these unvaccinated older adults using Latent Profile Analysis (LPA); (3) characterize these profiles by their socio-demographic and health attributes, and their association with HZ vaccination intention; and (4) explore the associations of these psychological profiles and physician recommendation with participants’ reported prior HZ vaccination intention and their likelihood of being in a specific profile.

## 2. Material and Methods

### 2.1. Study Design, Sampling, and Participants

A cross-sectional study was conducted from September 2024 to January 2025. A pilot study with 200 older adults informed minor wording adjustments to the questionnaire for clarity.

We employed a multistage sampling strategy across six major Chinese cities: Beijing, Qingdao, Hangzhou, Shenzhen, Chengdu, and Chongqing. Within each city, five to eight Community Health Centers (CHCs) were randomly selected. However, at the final stage of participant recruitment, well-trained interviewers used convenience sampling to consecutively approach individuals in waiting areas and other common areas within the selected CHCs. Potential participants were initially screened by CHC staff or trained interviewers for preliminary eligibility criteria: (1) aged 60 years or older, (2) had no history of HZ vaccination, and (3) provided written informed consent.

Regarding the response rate: Of the 13,754 individuals who passed the initial screening and were formally approached to participate in the study, 12,357 provided valid questionnaires after data screening for age and logical inconsistencies. This yielded a response rate of 96.9%. The high response rate reflects the fact that the denominator (13,754) only included individuals who had already met the key eligibility criteria (age and vaccination status) and were amenable to being approached in a healthcare setting. Data were collected via face-to-face interviews.

### 2.2. Questionnaire Design

A structured questionnaire collected data in three sections:

- Demographic and Health Characteristics: Included gender, age, income, education, occupation, city, chronic diseases, and self-reported health status.
- Vaccination History and Physician Recommendation: Assessed history of HZ, influenza, and pneumococcal vaccination. A key item measured whether participants had ever received a physician’s recommendation for the HZ vaccine (Yes/No).
- The 5C Scale for Vaccine Hesitancy: This 14-item scale measured the five psychological antecedents of vaccination on a 5-point Likert scale (1 = Strongly Disagree to 5 = Strongly Agree): Confidence (trust in vaccine safety/effectiveness), Complacency (low perceived disease risk), Constraints (practical barriers), Calculation (information seeking and risk–benefit analysis), and Collective Responsibility (willingness to vaccinate to protect others).

### 2.3. Reliability and Validity Assessment of Theoretical Framework

The measurement model’s validity was rigorously assessed. A Confirmatory Factor Analysis (CFA) confirmed the five-factor structure, demonstrating good model fit. To achieve the reported acceptable fit indices (χ^2^/df = 4.680, CFI = 0.936, TLI = 0.917, RMSEA = 0.074, SRMR = 0.079), minor modifications were made based on modification indices and theoretical considerations. Specifically, no items were deleted or rephrased from the original scale. Instead, correlated error terms were added between the following pairs of items, reflecting their potential semantic overlap or shared method variance:

- Within the Complacency dimension: Error term between Q4 (“The probability of getting diseases is low, so I do not need to get vaccinated.”) and Q5 (“Even if I get infected with a disease I can resist it, so I don’t need to be vaccinated.”). These two items both reflect a low perceived susceptibility and severity, indicating a strong conceptual overlap.
- Within the Constraints dimension: Error term between Q10 (“It was easy and took me a short time to get the vaccination.”) and Q11 (“I know the vaccination process in CHCs.”). This was justified as knowledge of the vaccination process (Q11) directly contributes to the perceived ease and time efficiency of getting vaccinated (Q10).

Convergent validity was established, with all Composite Reliability (CR) values exceeding 0.70 and Average Variance Extracted (AVE) values surpassing 0.50. Discriminant validity was also confirmed, as the square root of each construct’s AVE was greater than its inter-construct correlations. The scale demonstrated good internal consistency, with an overall Cronbach’s alpha of 0.868.

### 2.4. Statistical Analysis

Data suitability for factor analysis was confirmed (Kaiser–Meyer–Olkin = 0.885; Bartlett’s test of sphericity: *p* < 0.001). Latent Profile Analysis (LPA) was used to identify distinct subgroups based on the 5C dimension scores. Model selection was guided by multiple fit indices (AIC, BIC, sBIC), classification quality (Entropy), and statistical significance tests (BLRT, LMR), balanced with theoretical interpretability [22,23].

Chi-square tests were used to compare characteristics across latent profiles. A multinomial logistic regression model was then developed to identify factors associated with profile membership, controlling for confounders. Adjusted odds ratios (aORs) with 95% confidence intervals (CIs) were reported. Statistical significance was set at *p* < 0.05. All analyses were performed using SPSS version 26.0 and R version 4.2.0.

## 3. Results

### 3.1. Model Selection for Latent Profile Analysis (LPA)

We fitted LPA models with one to six classes (Table 1). While fit indices like AIC and BIC continued to improve up to the 6-class model, the Bootstrap Likelihood Ratio Test (BLRT) became non-significant (*p* = 1.00) when comparing the 5- and 6-class models. This suggested that adding a sixth class did not improve model fit. Therefore, we focused our evaluation on the four- and five-class models.

The transition from four to five classes yielded a substantial drop in both AIC and BIC, supporting the extraction of a fifth profile. To critically evaluate classification precision, we examined the average posterior probabilities for the five-class solution (Table S5). The diagonal values, representing the probability of correct class assignment, were 0.827, 0.709, 0.905, 0.880, and 0.658 for Profiles 1 to 5, respectively. Although the value for Profile 5 (0.658) indicates some degree of overlap with Profile 2, the overall entropy remained exceptionally high (0.96), suggesting strong classification certainty for the vast majority of the sample.

Ultimately, the 5-class solution was selected as it offered the optimal balance of statistical fit, overall class separation, and the most theoretically meaningful interpretation of vaccine hesitancy patterns within the 5C framework.

### 3.2. Psychological Profiles of the Latent Classes Based on the 5Cs

**Figure 1:**
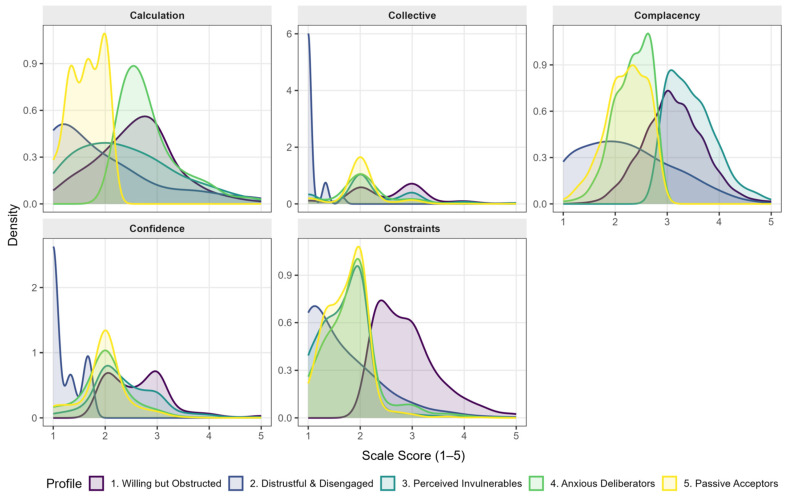
Kernel Density Distributions of 5C Scale Scores Across Latent Profiles. Note: The y-axis represents density (kernel density estimate). The area under each curve integrates to 1.0. Panels use independent y-scales for readability; therefore, peak heights are not comparable across dimensions.

The LPA conducted on the full sample of 12,357 unvaccinated older adults identified five distinct profiles, which differed significantly across all 5C dimensions (*p* < 0.001 for all). The profiles are described below (Table 2, Figure 1).

Profile 1: The Willing but Obstructed (*n* = 5509, 44.6%): The largest profile, characterized by the highest score on Constraints (M = 8.71) and high Collective Responsibility (M = 7.76), indicating a strong intention to vaccinate impeded by practical barriers.

Profile 2: The Distrustful and Disengaged (*n* = 2239, 18.1%): Defined by the lowest scores on Confidence (M = 3.60) and Collective Responsibility (M = 3.17). Their relatively low Calculation score (M = 5.75) suggests disengagement rooted in profound distrust.

Profile 3: The Perceived Invulnerable (*n* = 2217, 17.9%): Distinguished by the highest score on Complacency (M = 10.43), indicating a strong belief that HZ poses no personal threat.

Profile 4: The Anxious Deliberators (*n* = 916, 7.4%): The smallest profile, defined by the highest score on Calculation (M = 8.59), signifying a state of active, unresolved deliberation over vaccination pros and cons.

Profile 5: The Passive Acceptors (*n* = 1476, 11.9%): Characterized by the lowest scores on Calculation (M = 4.91) and Complacency (M = 6.64), reflecting a deference to medical authority that bypasses extensive personal risk analysis.

### 3.3. Demographic Characteristics of Participants and Profiles

No significant differences were found across profiles for age group, monthly income, chronic disease prevalence, or self-reported health status (Table S4).

However, significant differences emerged for several key characteristics. Gender distribution varied (χ^2^ = 12.748, *p* = 0.013), with more males in the “Perceived Invulnerable” class (47.7%) and more females in the “Distrustful and Disengaged” class (57.0%). Residence also showed a highly significant difference (χ^2^ = 90.061, *p* < 0.001). Education level was another distinguishing factor (χ^2^ = 20.010, *p* = 0.020), with the “Anxious Deliberators” showing a higher proportion of individuals with a high school education (30.2%).

The most pronounced differences were in vaccination history. Flu vaccine uptake during the 2024 season differed dramatically (χ^2^ = 889.224, *p* < 0.001), with the “Passive Acceptors” (52.0%) reporting high rates and the “Willing but Obstructed” (21.3%) reporting low rates. A similar pattern was observed for pneumococcal vaccination (χ^2^ = 845.101, *p* < 0.001). Finally, whether participants had ever received a physician’s recommendation for vaccination also differed significantly across the classes (χ^2^ = 10.487, *p* = 0.033). The “Distrustful and Disengaged” class reported the highest proportion of having received this type of recommendation (17.6%).

### 3.4. Associations Between Latent Profiles and Covariates

Multivariate logistic regression identified factors associated with profile membership, using the “Passive Acceptors” as the reference group (Table S6). Covariates were selected based on their established associations with vaccine attitudes and behaviors in the previous literature [16]. The full model included demographic characteristics (city of residence, education level), vaccination history (flu and pneumococcal vaccines), and external influences (physician’s recommendation). Prior to the analysis, multicollinearity was assessed using the Variance Inflation Factor (VIF). All VIF values were below 1.7, indicating that multicollinearity was not a concern.

Demographics: Compared to Chongqing residents, those from Shenzhen (aOR = 0.727, *p* = 0.002) and Hangzhou (aOR = 0.804, *p* = 0.034) were less likely to be “Willing but Obstructed.” Lower education levels were associated with higher odds of being in the “Distrustful and Disengaged” and “Anxious Deliberators” profiles compared to those with a bachelor’s degree or higher.

Vaccination History: Not having taken the flu vaccine was associated with a threefold increased likelihood of being in the “Willing but Obstructed” profile (aOR = 3.053, *p* < 0.001). Similarly, not having taken the pneumococcal vaccine increased the odds of belonging to the “Willing but Obstructed” (aOR = 2.261, *p* < 0.001), “Distrustful and Disengaged” (aOR = 1.257, *p* = 0.002), and “Perceived Invulnerable” (aOR = 1.750, *p* < 0.001) profiles. Physician’s recommendation was not significantly associated with membership in any profile.

### 3.5. Dissecting the Nature of Constraints in the “Willing but Obstructed” Profile

To resolve the paradox that income was not a significant predictor of profile membership while “Constraints” defined the largest profile, we analyzed self-reported reasons for non-vaccination within this group, stratified by income (Table 3). A significant inverse association emerged between income and citing the “High cost of the vaccine” (*p* = 0.002). Lower-income individuals were also more likely to report “Perceived ineffectiveness” (*p* = 0.001), while higher-income individuals more often cited “Concerns about side effects” (*p* = 0.042) and “Distrust of commercial motives” (*p* = 0.023).

## 4. Discussion

This study successfully identified five heterogeneous profiles related to HZ vaccination attitudes and intentions among unvaccinated older Chinese adults. Our findings demonstrate that profile membership is significantly associated with residence, education, and past vaccination behaviors, revealing a complex interplay of psychological and contextual factors among this specific population.

### 4.1. Interpretation of the Latent Profiles

The five profiles provide a nuanced understanding that moves beyond a monolithic view of vaccine hesitancy as it pertains to individuals who have not yet received the HZ vaccine. The largest profile, the “Willing but Obstructed” (44.6%), exemplifies the classic “intention-behavior gap” [18,19]. Our analysis revealed a strong association between household income and specific constraints within this profile: while not a direct predictor, the perception of “high cost” was overwhelmingly concentrated in the low-income group. This highlights a significant financial barrier that, from a behavioral economics perspective, can be particularly salient due to present bias (the immediate, certain cost outweighs the future, uncertain health benefit) and loss aversion (the tangible financial outlay is perceived as a greater loss than the potential future gain of avoiding illness) [22]. This indicates that socioeconomic status is closely intertwined with tangible structural obstacles, such as financial barriers, to hinder vaccination intentions in this willing group [20,21].

A counter-intuitive finding emerged from the “Distrustful and Disengaged” (18.1%) profile. Despite having the lowest Confidence, they reported the second-highest uptake of flu and pneumococcal vaccines. This paradox could be explained by several factors: their distrust may be context-specific (e.g., directed at newer vaccines like HZ but not established ones like flu/pneumococcal) or other factors, like strong physician relationships, might override their personal beliefs for established vaccines [23,24]. However, as these interpretations remain speculative, several alternative explanations must be considered. First, recall bias in self-reported vaccination history may be present, as participants might over-report past uptake due to social desirability. Second, cohort effects may play a role, as older adults might have adhered to long-standing vaccination routines established earlier in life. Third, the differential impact of public health campaigns and the superior accessibility of influenza and pneumococcal vaccines—compared to the HZ vaccine—may explain this discrepancy [25]. Collectively, these findings challenge the assumption that low confidence invariably leads to low uptake across all vaccine types.

The “Perceived Invulnerable” (17.9%) profile’s hesitancy is rooted in high Complacency, aligning with the Health Belief Model (HBM) [26]. Their low perceived risk, not a lack of vaccine confidence, is the primary driver. From a behavioral economics standpoint, this low perceived risk (despite objective epidemiological data) could be influenced by optimism bias (believing negative events are less likely to happen to oneself) or the availability heuristic (lack of direct experience with HZ leading to underestimation of personal risk) [27]. This suggests that risk communication focusing on personal susceptibility and disease severity may be particularly effective for this group [8,28,29].

The “Anxious Deliberators” (7.4%), with their highest Calculation score, suffer from “analysis paralysis,” becoming overwhelmed while weighing vaccination pros and cons [30]. This group does not need more data; they may benefit from clear, personalized guidance from a trusted source to facilitate a decision [31,32].

Finally, the “Passive Acceptors” (11.9%) represent a pathway to vaccination characterized by deference to medical authority, reinforcing the pivotal role of healthcare providers [33].

### 4.2. Predictors of Profile Membership

The association with residence suggests that local public health environments and healthcare accessibility may shape psychological profiles [32]. The link between lower education and membership in the “Distrustful” and “Anxious” profiles aligns with health literacy literature, indicating that challenges in processing health information can contribute to both distrust and indecision [31,33]. Unsurprisingly, past vaccination behavior was the strongest predictor, further characterizing the distinct nature of each profile.

### 4.3. Theoretical and Practical Implications

Theoretically, this study demonstrates the value of applying LPA to the 5C model, showing that psychological antecedents operate in combination to form distinct profiles. Practically, our findings suggest the utility of moving away from “one-size-fits-all” campaigns to tailored, profile-based strategies. While these recommendations should be interpreted as hypotheses for future testing rather than definitive policy directives, they offer valuable implications for engaging unvaccinated older adults:

(1) For the “Willing but Obstructed”: Interventions could consider being equity-focused. Policies like means-tested subsidies, free vaccination programs for low-income seniors, and mobile clinics may be crucial to dismantle the structural barriers that perpetuate health inequality.
(2) For the “Distrustful and Disengaged”: Trust-building appears paramount. Interventions could leverage trusted community leaders and primary care physicians for empathetic and transparent communication, as mass media campaigns might be less effective for this group.
(3) For the “Perceived Invulnerable”: Communication strategies could focus on visceral messaging about personal risk and the severity of HZ and its complications, such as PHN.
(4) For the “Anxious Deliberators”: A key goal could be to reduce cognitive burden. Clear decision aids and facilitated conversations with trusted healthcare providers may help resolve their indecision.

Beyond psychological antecedents, our findings also resonate with principles from behavioral economics, particularly concerning the interplay of perceived costs and benefits in health decision-making [34,35]. The economic landscape of HZ vaccination in China has evolved with the market entry of two distinct vaccines: the recombinant zoster vaccine in 2020 and the domestic live attenuated zoster vaccine in 2023. These vaccines are priced at approximately USD 193 and USD 225 per dose, respectively, and are typically self-funded [36,37]. The cost–benefit proposition remains challenging for many older adults when weighed against a relatively low perceived risk of infection (5–6 per 1000 person-years). From a bounded rationality perspective, an individual’s decision may be influenced by cognitive biases such as present bias (weighting immediate costs over future health gains) and optimism bias (underestimating personal risk) [27,34]. Addressing these drivers is essential for intervention design. For instance, reframing vaccination as a “health investment” or emphasizing the potential “loss” of quality of life leverages loss aversion to nudge individuals toward proactive choices [29,35]. While our study did not empirically test these interventions, it provides a robust theoretical foundation for developing profile-specific nudge-based strategies in future public health efforts.

### 4.4. Strengths and Limitations

This study’s strengths include its large, multi-city sample and the robust application of the 5C framework with LPA. However, several limitations should be considered. First, the cross-sectional design precludes causal inference and the ability to predict actual future vaccination behavior from profile membership. We were also unable to longitudinally track whether profile membership translates into subsequent vaccination uptake. Second, the study population was restricted to older adults who had no prior history of HZ vaccination. This means our findings describe psychological predispositions and intentions among the unvaccinated. Third, reliance on self-reported data is subject to potential biases, including social desirability bias, particularly in face-to-face interview settings, where participants might provide socially acceptable responses. Fourth, while a multistage design was used for city and CHC selection, the convenience sampling employed for participant recruitment within CHCs likely introduced selection bias. This approach may potentially limit the generalizability of our findings to individuals less connected to healthcare or residing in rural areas. Future studies should aim for more probability-based sampling strategies to enhance representativeness across a broader spectrum of older adults. Finally, other unmeasured variables, like the quality of patient–provider interactions, may also play a role in shaping vaccination attitudes.

## 5. Conclusions

This study sheds light on the diverse psychological profiles related to HZ vaccination attitudes and intentions among previously unvaccinated older adults, thereby contributing to a more nuanced understanding of factors often grouped under the broad concept of “vaccine hesitancy” within this specific population. Moving from a ‘one-size-fits-all’ to a profile-based approach may allow public health efforts to be tailored with greater precision and efficacy, ultimately contributing to efforts to address the significant gap between vaccination intention and uptake among unvaccinated older adults.

## Figures and Tables

**Table 1:** Model Fit Indices for Latent Class Analysis.

Number of Classes	AIC *	BIC *	Entropy	Smallest Class Proportion	BLRT * *p*-Value
1	310,433	310,508	1.00	100%	-
2	298,344	298,464	0.75	40.0%	<0.01
3	293,610	293,775	0.80	15.0%	<0.01
4	291,851	292,061	0.82	6.0%	<0.01
5	278,673	278,928	0.96	5.0%	<0.01
6	277,674	277,974	0.95	4.0%	1.00

* AIC = Akaike Information Criterion; BIC = Bayesian Information Criterion; BLRT = Bootstrap Likelihood Ratio Test.

**Table 2:** Mean Scores of 5C Dimensions and Characteristics of the Five Latent Profiles.

5C Dimension	Total (*N* = 12,357)	Willing but Obstructed (*n* = 5509, 44.6%)	Distrustful and Disengaged (*n* = 2239, 18.1%)	Perceived Invulnerable (*n* = 2217, 17.9%)	Anxious Deliberators (*n* = 916, 7.4%)	Passive Acceptors (*n* = 1476, 11.9%)	*p*-Value
Confidence	6.64 ± 2.29	7.96 ± 1.81	3.60 ± 0.83	7.15 ± 1.93	6.01 ± 1.46	5.94 ± 1.38	<0.001
Calculation	7.02 ± 2.66	7.77 ± 2.29	5.75 ± 3.09	7.18 ± 2.80	8.59 ± 1.78	4.91 ± 0.98	<0.001
Constraints	6.71 ± 2.50	8.71 ± 1.79	4.91 ± 2.18	5.07 ± 1.41	5.49 ± 1.60	5.20 ± 1.19	<0.001
Complacency	8.57 ± 2.31	9.38 ± 1.73	6.66 ± 2.63	10.43 ± 1.36	6.99 ± 1.01	6.64 ± 1.15	<0.001
Collective Responsibility	6.39 ± 2.61	7.76 ± 2.36	3.17 ± 0.45	6.61 ± 2.44	6.11 ± 1.76	5.98 ± 1.38	<0.001

**Table 3:** Association between Monthly Income and Reported Barriers among Members of the “Willing but Obstructed” Profile (*N* = 5509).

Reason for Non-Vaccination	Low Income (≤2500 CNY) (*n* = 1632)	Medium Income (2501–5000 CNY) (*n* = 2417)	High Income (>5000 CNY) (*n* = 1460)	Total	*p*-Value
	*n* (%)				
**Reasons Reflecting Constraints**					
High cost of the vaccine	433 (26.5)	586 (24.2)	307 (21.0)	1326 (24.1)	0.002
Inconvenient time or location	174 (10.7)	235 (9.7)	156 (10.7)	565 (10.3)	0.514
**Reasons Reflecting Lack of Confidence**					
Concerns about side effects/adverse reactions	259 (15.9)	401 (16.6)	279 (19.1)	939 (17.0)	0.042
Perceived ineffectiveness of the vaccine	299 (18.3)	356 (14.7)	205 (14.0)	860 (15.6)	0.001
Influence of negative media/online information	82 (5.0)	141 (5.8)	83 (5.7)	306 (5.6)	0.527
Distrust of commercial motives/profiteering	81 (5.0)	147 (6.1)	107 (7.3)	335 (6.1)	0.023
**Reason Reflecting Lack of Collective Responsibility**					
Lack of vaccination among peers/family	473 (29.0)	630 (26.1)	458 (31.4)	1561 (28.3)	0.001

## Data Availability

The analysis code for this study is available on GitHub: [https://github.com/daisy0113ning-a11y/vaccine-hesitancy-lpa/tree/main (accessed on 25 May 2024)]. The dataset generated and analyzed during the current study is not publicly available due to participant privacy restrictions but is available from the corresponding author on reasonable request.

## Supplementary Materials

The following supporting information can be downloaded at: https://www.mdpi.com/article/10.3390/vaccines14040331/s1, Table S1: Coding of independent variables used in regression models; Table S2: The modified 5C scale of vaccine hesitancy; Table S3: Reliability and validity assessment of the modified 5C scale; Table S4: Discriminant Validity Assessment—Fornell–Larcker Criterion; Table S5: Average Posterior Probabilities for the Five-Class Solution; Table S6: Associations between the five latent profiles and demographic characteristics from the multivariate logistic regression model.
