# Supplementary material for: Profiles of Hesitancy Toward the Herpes Zoster Vaccine Among Older Adults in China: A Latent Profile Analysis
Source: Vaccines (Basel). 2026 Apr 8;14(4):331. doi: 10.3390/vaccines14040331 (PMC13119683; doi:10.3390/vaccines14040331)
Supplement: Supplementary file 1 [file vaccines-14-00331-s001.zip › vaccines-4204695-supplementary.pdf]

Supplementary Material

Table S1. Coding of independent variables used in regression models.

Independent	Assignment
Monthly income	≤2500 CNY = 1; 2501-5000 CNY = 2; >5000 CNY = 3
Educational level	Primary school and below = 1; Middle school = 2; High school = 3; Bachelor's degree and above = 4
Age group	60-64 = 1; 65-69 = 2; 70-74 = 3; ≥75 = 4
Gender	Male = 1; Female = 2
Self-reported health	Poor = 1; Fair = 2; Good = 3; Very good = 4
Chronic diseases	Yes = 1; No = 0
Residence	Beijing = 1; Chengdu = 2; Hangzhou = 3; Qingdao = 4; Shenzhen = 5; Chongqing = 6
Have taken the flu vaccine during the 2024 flu season	Yes = 1; No = 0
Have taken the pneumococcal vaccine	Yes = 1; No = 0
Physicians' recommendation	Yes = 1; No = 0

Table S2. The modified 5C scale of vaccine hesitancy.

Dimension	Questions
Confidence	Q1. I think that vaccination is safe.
	Q2. I trust physicians' recommendation of vaccination to benefit my health.
	Q3. I trust the vaccination provided by the government to protect old adults.
Complacency	Q4. The probability of getting diseases is low, so I do not need to get vaccinated.
	Q5. Even if I get infected with a disease I can resist it, so I don't need to be vaccinated.
Calculation	Q6. I carefully considered the efficacy and risks of the vaccines.
	Q7. I got the information of vaccins seriously before vaccination.
	Q8. I took the initiative to consult a physicians for vaccination advice.
Constraints	Q9. It was easy to get the information of vaccines.
	Q10. It was easy and took me a short time to get the vaccination.
	Q11. I know the vaccination process in CHCs.
Collective Responsibility	Q12. I think that vaccination can protect not only me but also others from infectous.
	Q13. I think getting vaccinated can reduce the spread of diseases among the population.
	Q14. I am willing to protect population with low immunity by getting vaccinated.

Each item in five sections was assessed using a 5-point Likert, ranging from 1 (strongly disagree, completely impossible, etc.) to 5 (strongly agree, completely possible, etc.).

Table S3. Reliability and validity assessment of the modified 5C scale.

Dimension items	Cronbach's α	AVE	CR
Confidence	0.857	0.696	0.894
Complacency	0.681	0.565	0.751
Calculation	0.615	0.503	0.706
Constraints	0.715	0.508	0.719
Collective Responsibility	0.959	0.888	0.956

Table S4. Discriminant Validity Assessment - Fornell-Larcker Criterion.

Construct	1	2	3	4	5
1. Confidence	0.834				
2. Complacency	-0.523**	0.752			
3. Calculation	-0.296**	0.314**	0.784		
4. Constraints	-0.411**	0.306**	0.308**	0.846	
5. Collective Responsibility	0.692**	-0.397**	-0.276**	0.442**	0.979

Note: $^{*}p < 0.05$, $^{**}p < 0.01$

Table S5. Average Posterior Probabilities for the Five-Class Solution.

Profile	1	2	3	4	5
1. Willing but Obstructed	**0.827**	0.091	0.019	0.010	0.054
2. Distrustful and Disengaged	0.021	**0.709**	0.029	0.041	0.199
3. Perceived Invulnerable	0.005	0.045	**0.905**	0.000	0.044
4. Anxious Deliberators	0.004	0.093	0.000	**0.880**	0.023
5. Passive Acceptors	0.017	0.264	0.051	0.011	**0.658**

Table S6. Associations between the five latent profiles and demographic characteristics from the multivariate logistic regression model.

Variables	Willing but Obstructed (N=5,509)			Distrustful and Disengaged (N=2,239)			Perceived Invulnerable (n=2,217)			Anxious Deliberators (n=916)		
	aOR	95 % CI	p-value	aOR	95 % CI	p-value	aOR	95 % CI	p-value	aOR	95 % CI	p-value
**Gender**												
Male	0.907	0.805-1.022	0.109	0.885	0.775-1.012	0.074	1.012	0.885-1.158	0.857	0.928	0.784-1.097	0.379
Female		Ref.			Ref.			Ref.			Ref.	
**Residence**												
Beijing	0.908	0.732-1.127	0.382	1.099	0.865-1.396	0.441	0.791	0.618-1.012	0.062	0.877	0.653-1.178	0.383
Chengdu	0.895	0.728-1.101	0.294	1.017	0.807-1.281	0.887	0.890	0.705-1.123	0.326	0.604	0.447-0.817	0.001
Hangzhou	0.804	0.657-0.983	0.034	1.035	0.828-1.295	0.760	0.863	0.687-1.083	0.203	0.833	0.633-1.096	0.192
Qingdao	0.840	0.685-1.029	0.093	0.955	0.760-1.199	0.691	0.803	0.637-1.013	0.064	0.711	0.534-0.946	0.019
Shenzhen	0.727	0.592-0.893	0.002	0.786	0.622-0.994	0.044	1.047	0.834-1.313	0.694	0.868	0.658-1.146	0.318
Chongqing		Ref.			Ref.			Ref.			Ref.	
**Education Level**												
Primary school and below	1.051	0.864-1.127	0.622	1.134	0.908-1.415	0.267	0.997	0.801-1.242	0.981	1.356	1.016-1.809	0.039
Middle school	1.163	0.728-1.403	0.114	1.208	0.978-1.492	0.080	0.986	0.799-1.218	0.897	1.291	0.977-1.705	0.072
High school	1.182	0.977-1.429	0.085	1.275	1.030-1.579	0.026	1.044	0.844-1.292	0.690	1.687	1.283-2.220	<0.001
Bachelor's degree and above		Ref.			Ref.			Ref.			Ref.	
**Have taken the flu vaccine during the 2024 flu season**												
Yes		Ref.			Ref.			Ref.			Ref.	

No	3.053	2.677-3.482	<0.001	0.752	0.646-0.874	<0.001	1.780	1.485-2.062	<0.001	1.078	0.850-1.368	0.534
**Have taken the pneumococcal vaccine**												
Yes		Ref.			Ref.			Ref.			Ref.	
No	2.261	1.954-2.617	<0.001	1.257	1.087-1.454	0.002	1.750	1.485-2.062	<0.001	1.060	0.873-1.287	0.556
**Physicians' recommendation**												
Yes		Ref.			Ref.			Ref.			Ref.	
No	0.991	0.839-1.171	0.915	0.850	0.708-1.021	0.082	1.080	0.893-1.306	0.428	1.078	0.850-1.368	0.534
